# *LOXL1* gene variants and their association with pseudoexfoliation glaucoma (XFG) in Spanish patients

**DOI:** 10.1186/s12881-015-0221-y

**Published:** 2015-08-31

**Authors:** Lydia Álvarez, Montserrat García, Héctor González-Iglesias, Julio Escribano, Pedro P. Rodríguez-Calvo, Luis Fernández-Vega, Miguel Coca-Prados

**Affiliations:** Fundación de Investigación Oftalmológica, Instituto Oftalmológico Fernández-Vega, Avenida Doctores Fernández-Vega, 34, Oviedo, 33012 Spain; Laboratorio de Genética Molecular Humana, Facultad de Medicina/Instituto de Investigación en Discapacidades Neurológicas (IDINE), Universidad de Castilla-La Mancha, Albacete, 02006 Spain; Department of Ophthalmology and Visual Science, Yale University School of Medicine, New Haven, CT 06510 USA

## Abstract

**Background:**

*LOXL1* gene is the most important genetic risk factor known so far for pseudoexfoliation glaucoma (XFG). Our purpose was to evaluate the potential association of individual genetic variants of the *lysyl oxidase-like 1* (*LOXL1*) gene and haplotypes with XFG in Spanish patients.

**Methods:**

Blood samples were collected from a total of 105 Spanish patients with XFG and 200 healthy controls. The entire *LOXL1* gene along with the promoter, coding and non-coding regions including the 5´- and 3´-untranslated regions, were sequenced using next-generation sequencing in 99 XFG patients. SNPs rs16958477 (promoter), rs1048661 (exon 1), rs3825942 (exon 1), rs2165241 (intron 1) and rs3522 (exon 7) in *LOXL1* were genotyped by restriction fragment-length polymorphism (RFLP) in all Spanish control participants and in six additional XFG patients, and a case–control association study was performed.

Comparisons of the allelic and genotypic frequencies were performed using standard χ^2^ test with Bonferroni and Pearson corrections. Logistic regression analyses were permormed using Sigmaplot v11. Haplotypes frequencies were performed using HaploView 4.0.

**Results:**

Sequencing of the *LOXL1* gene in XFG participants identified a total of 212 SNPs, of which 49 exhibited allelic frequencies with significant differences between cases and controls, and 66 were not previously described. The allele frequencies of SNPs rs16958477, rs1048661, rs3825942, rs2165241, were significantly associated with an increased risk for XFG, however the SNP rs3522 was not.

The haplotype frequencies of SNPs rs16958477, rs1048661, rs3825942 and rs2165241 and their association with XFG indicated that the CGGT haplotype, containing all four risk alleles, and the AGGT haplotype, which carries the protective allele of rs16958477 and three risk alleles of the other three SNPs, were significantly associated with XFG (*p* = 4.5×10^−6^, and *p* = 8.8×10^−6^), conferring more than 2-fold increased disease susceptibility.

**Conclusions:**

SNPs of the *LOXL1* gene are associated with XFG in the Spanish population. This information adds new support to the distinct risk association frequencies of *LOXL1* alleles with XFG in Western European and Asian populations. Identification and validation of additional SNPs along the entire *LOXL1* gene of XFG cases may provide insightful information on their potential role in the pathogenesis of this disease.

**Electronic supplementary material:**

The online version of this article (doi:10.1186/s12881-015-0221-y) contains supplementary material, which is available to authorized users.

## Background

Pseudoexfoliation glaucoma (XFG) is the most common form of secondary open-angle glaucoma and develops in the context of pseudoexfoliation syndrome (XFS). XFS is an age-related systemic disorder of the extracellular matrix characterized by production and progressive deposition of abnormal microfibrillar material in ocular and extraocular tissues [1, 2]. The prevalence of XFS varies widely throughout the world. In people aged 60 or above, the prevalence of XFS is up to 25 % in the Scandinavian peninsula [2], 5–15 % in Central European populations [3, 4], 4.4 % in Japanese [5], 1.6–3 % in Caucasian Americans, 0.4 % in African-Americans [6, 7], <1 % in Chinese [8, 9], and 0 % in Greenland Eskimos [10, 11]. Relatively high prevalence (18.9 %) has been reported in a population of northwestern Spain [12].

The conversion rate of XFS to XFG has been estimated to be 44 % over 15 years [13, 14]. In XFG patients, the deposition of exfoliation material in the anterior segment of the eye causes resistance to the outflow of aqueous humor, leading to elevated intraocular pressure and consequent glaucomatous optic neuropathy [15, 16]. At the clinical level, XFG is characterized by rapid progression of glaucomatous optic nerve damage, poor response to medical treatment, and worse prognosis than primary open-angle glaucoma [2].

Extensive evidence indicates that genetic factors predispose individuals to XFS/XFG, including increased risk in certain racial and ethnic groups, and family aggregation, especially in twins [10, 17–19]. Moreover, in a genome-wide association study (GWAS) performed in 2007 in Scandinavian populations, Thorleifsson et al. found a strong association between XFS/XFG and three single nucleotide polymorphisms (SNPs) on the lysyl oxidase-like 1 (*LOXL1*) gene on chromosome 15q24.1 [20]. One of these SNPs, rs2165241, is a non-coding SNP located in intron 1 of *LOXL1*. The other two SNPs, located in exon 1, are nonsynonymous coding SNPs, rs1048661 (resulting in an arginine to leucine amino-acid change, R141L) and rs3825942 (resulting in a glycine to aspartic amino-acid change, G153D). The allele T for the intronic SNP, and the allele G for both coding SNPs are associated with a higher risk of XFS and XFG in the studied population. Thus, the LOXL1 protein containing [Arg141-Gly153] is the one associated with the highest risk for XFG in this population.

Since this original discovery was reported, the association of *LOXL1* SNPs with XFS/XFG has been studied in populations of diverse geographic origins, including USA, South America, Australia, Europe, Middlle East, Africa, and Asia (Table 1), confirming the *LOXL1* gene as the most important genetic risk factor known so far for these pathologies. However, the frequencies of risk alleles for the above described *LOXL1* SNPs were also high in controls and vary among different populations within the same country, and among different ethnic groups. For example, the T allele of rs1048661 was found significantly associated with XFS/XFG in certain populations from China [21], Japan [22–27] and Korea [28, 29], but not in other populations from China [30] and India [31]. In contrast, in a recent study with XFS/XFG cases in South India it was found the G allele of rs1048661 as the susceptibility variant [32]. Similarly, the intronic SNP (rs2165241) was found with no association with XFG in a Japanese population [22] while other studies carried out with Chinese [21], Japanese [26, 27] and Korean subjects [28, 29] suggested the alternative C allele as the risk variant. Regarding the SNP rs3825942, studies performed in a South African black population have reported the allele A, instead of G, as the susceptibility factor [33, 34]. In contrast, the alternative allele G, has been identified as the risk variant in other XFS/XFG populations (Table 1). Most recently, a study conducted with a Spanish population showed the lack of association between the SNP rs10488661 and XFS/XFG [35]. This is in contrast to what it has been found for most caucasian populations (Table 1). All together, the association of these SNPs with XFG may not be strong enough and other genes and/or other variants in the *LOXL1* gene may contribute to the risk of XFG.

**Table 1:** Comparative data of *LOXL1* risk alleles in different populations with XFS/XFG

Population (Reference)	rs1048661		rs3825942		rs2165241	
	Risk allele	*p* value	Risk allele	*p* value	Risk allele	*p* value
Spanish (present study)	G	8.47×10^−8^	G	2.54×10^−8^	T	6.30×10^−16^
Spanish [35]	No assoc.	0.19	G	3.36×10^−5^	T	2.50×10^−4^
Icelandic [20]	G	1.80×10^−6^	G	4.10×10^−9^	T	4.3×10^−12^
Swedish [20]	G	2.70×10^−7^	G	9.10×10^−14^	T	3.1×10^−17^
Finnish [47]	G	1.47×10^−5^	G	4.82×10^−10^	T	7.36×10^−14^
Austrian [57]	G	2.55×10^−7^	G	5.76×10^−15^	NA	NA
German [48]	G	4.32×10^−16^	G	1.21×10^−11^	T	6.77×10^−30^
Italian [48]	G	0.0009	G	1.66×10^−18^	T	2.19×10^−10^
Polish [53]	No assoc.	0.09	G	0.0047	T	0.021
Greek [52]	No assoc.	0.490	G	1.56×10^−5^	T	0.016
Greek [58]	G	0.0045	G	8.65×10^−4^	NA	NA
Greek [59]	No assoc.	0.1226	G	0.0003	NA	NA
American and European [51]	G	7.74×10^−9^	G	3.10×10^−17^	T	4.85×10^−24^
American [49]	G	0.0222	G	0.0194	T	0.001
American [50]	G	0.0031	G	1.3×10^−13^	T	1.50×10^−5^
American [60]	NA	NA	G	4.53×10^−5^	T	1.09×10^−8^
Australian [61]	G	8.49×10^−4^	G	7.83×10^−5^	NA	NA
Turkish [62]	G	7.08×10^−7^	G	5.8×10^−16^	NA	NA
Pakistani [63]	G	1×10^−7^	G	1×10^−7^	NA	NA
Saudi Arabian [36]	G	0.0056	G	5×10^−6^	NA	NA
Mexican [64]	No assoc.	0.71	G	0.0019	T	1×10^−5^
Uygur [65]	G	0.013	G	<0.001	T	<0.001
Chinese [30]	No assoc.	0.142	G	0.0018	NA	NA
Chinese (Uygur) [21]	T	6.95×10^−11^	G	8×10^−4^	C	0.01
Indian [31]	No assoc.	0.156	G	0.0001	NA	NA
South Indian [32]	G	4.28×10^−5^	G	4.68×10^−30^	T	1.98×10^−15^
Japanese [22]	T	7.70×10^−18^	G	4.10×10^−4^	No assoc.	NA
Japanese [23]	T	3×10^−19^	G	1.4×10^−5^	NA	NA
Japanese [24]	T	<0.0001	G	<0.0001	NA	NA
Japanese [25]	T	<1×10^−8^	G	7×10^−8^	NA	NA
Japanese [26]	T	6.41×10^−48^	G	1.30×10^−11^	C	2.31×10^−7^
Japanese [27]	T	5.65×10^−33^	G	2.42×10^−22^	C	7.87×10^−7^
Korean [28]	T	2.13×10^−12^	G	9.12×10^−6^	C	2.59×10^−3^
Korean [29]	T	5.74×10^−12^	G	0.0003	C	0.0011
Black South African [33]	G	0.00106	A	<0.00001	NA	NA
Black South African [34]	G	1.7×10^−5^	A	5.2×10^−13^	NA	NA

*XFS*; pseudoexfoliation syndrome, *XFG*; pseudoexfoliation glaucoma, *NA*; data not available,* No assoc.*; no association

The complete *LOXL1* coding region has been sequenced in different populations searching for new SNPs variants [32, 34, 36, 37]. Two recent studies, carried out with South Indian and Mexican subjects, have identified exon 1 rs41435250 SNP, as a new risk factor [32, 37]. The latter study suggested the possibility of a *LOXL1* intragenic epistatic effect between SNPs rs41435250 and rs2165241 [37]. However, this finding was not replicated in South African subjects [34]. Furthermore, this SNP was found to be monoallelic in a sample of Saudi Arabian XFG cases and controls [36].

In the absence of a definitive association within variants of *LOXL1* coding region and XFG, regulatory regions are now the focus of attention. Fan et al. found an association between the allele A of the *LOXL1* promoter variant rs16958477, located 659 base pairs upstream of the transcription start site of the gene and XFS, in a large caucasian North American cohort. In addition, haplotype analysis has shown that the risk allele of this variant was significantly correlated with the previously identified risk alleles of the SNPs rs1048661 and rs3825942, suggesting that these variants are associated with the disease risk because of linkage disequilibrium with the rs16958477 risk allele [38]. Promoter studies showed the SNP rs16958477 as a transcriptionally relevant SNP since its C allele had significantly greater activity than the allele A [39]. In contrast, the SNP rs16958477 did not show association with XFG in a black South African population [34].

The present study was designed to: i) seek genetic variants spanning the whole gene region, including promoter, coding, noncoding and 5´- 3´untranslated regions, in XFG cases in Spanish subjects; and ii) analyze the association of the most important SNPs described to date in the *LOXL1* gene with XFG in a Spanish population.

## Methods

### Study subjects

The present case–control study included 105 unrelated native Spanish patients diagnosed with XFG and 200 age- and ethnically matched healthy people as controls recruited at the Instituto Oftalmológico Fernández-Vega, in Oviedo, Spain. Most subjects involved in the study (75 and 66 % of XFG and controls, respectively) originated in the Northernwestern regions of Spain including Galicia, Asturias and Cantabria. Complete ophthalmic examinations were performed for both, patients and controls. Subjects with XFG exhibited deposits of exfoliative material on the anterior lens surface and/or iris during slit-lamp examination, in one or both eyes. In addition, these patients showed the characteristic optic-disc damage (e.g., vertical cup-to-disc ratio >0.3, thin or notched neuroretinal rim, or disc hemorrhage) with the corresponding characteristic changes in the visual field. Control subjects were selected from patients undergoing cataract surgery who did not present with signs of glaucoma or exfoliative material. Of note, most glaucoma patients (94.3 %) also had cataracts. No subjects involved in this study presented with other relevant ocular pathologies such as retinopathies, or maculopathies. Considering that XFG is a late-onset disorder, to avoid possible misclassification, only people aged 60 or above were recruited as controls. The number of subjects, gender, mean and range of ages in each group are reported in Table 2.

**Table 2:** Demographic characteristics of XFG patients and controls

Study population (n)	Age (mean ± SD)	Age range	Gender (female/male)
Controls (200)	71.42 ± 7.64	60-92	130 (65 %)/70
XFG (105)	73.46 ± 7.89	52-92	56 (53.3 %)/49

*XFG*; pseudoexfoliation glaucoma, *n*; number of subjects, *SD*; standard desviation

The study adheres to the tenets of the Declaration of Helsinki on Biomedical Research Involving Human Subjects, and was approved by the Clinical Research Ethics Committee at the Hospital Universitario Central de Asturias (Oviedo, Spain). All participants signed an informed consent.

### Blood collection and DNA isolation

Peripheral blood samples from each participant enrolled in this study were collected in 6 mL K2E K2EDTA tubes coated with EDTA, which blocks the coagulation cascade (Vacuette, Madrid, Spain). Tubes were stored at −20 °C until use. Genomic DNA was prepared from the blood samples of all studied subjects using a commercial DNA extraction kit (FlexiGene DNA Kit; Qiagen, Hilden, Germany) according to the manufacturer’s protocol.

### *LOXL1* SNPs discovery study

A 39,230 bp genomic region (Chr15: 74211419–74250648), contained the complete *LOXL1* gene (25,690 bp), and 5´ (7370 bp) and 3´ (6170 bp) flanking untranslated (UT) regions, was sequenced from 99 XFG participants. The 39.2-Kb DNA region was amplified by polymerase chain reaction (PCR) into 4 amplicons of 10-Kb-long, using 4 sets of commercial primers (Applied Biosystems, Langen, Germany).

SeqTarget LongRange PCR Kit (Applied Biosystems, Langen, Germany) was used for all the PCR amplifications (for further information about the PCR conditions see Additional file 1). Amplicons 1, 2 and 3 were directly purified with QIAquick PCR Purification Kit (Qiagen, Hilden, Germany), whereas amplicon 4 was purified from low-melt agarose gels with UltraClean 15 DNA Purification Kit (Mo Bio Laboratories, Carlsbad, CA) or QIAEX II Gel Extraction Kit (Qiagen, Hilden, Germany), in both cases according to manufacturer’s protocols.

The purified amplicons were quantified on a spectrophotometer (Picodrop Ltd., Cambridge, UK) and products obtained from the same sample were pooled in equimolar amounts. Next, 1.2 μg DNA of each pool was fragmented to a 300 bp segments in solution using a Bioruptor sonicator (Diagenode, Belgium) with the following settings: 30 s ‘on’, 30 s ‘off’ on power setting ‘high’. Library preparation was performed following “TruSeq® DNA Sample Preparation Protocol” (Illumina Inc., CA, USA). Libraries between 300 and 500 bp were selected, which correspond to inserts sizes between 200 and 400 bp (average 300 bp). Pools of 18 libraries were sequenced in each lane (12 pM final concentration of the pool) of a flow-cell (Illumina Inc.) using the platform HiScanSQ (for more details see Additional file 1). DNA fragmentation, library preparation and sequencing were carried out at the Genome Analysis Platform (CIC bioGUNE, Bilbao, Spain). Data analysis was carried out at Dreamgenics (Dreamgenics, Llanera, Spain). In brief, single-end reads originated from sequencing were splitted into individual sample files by using unique specific barcodes, and then alligned using the bwa algorithm (0.6.1 version) against the complete human genome reference sequence (GRCh37/hg19). The resulting alignment was then converted and sorted into a BAM file using Samtools-0.1.18 software package. Samtools and in-house Perl and Java algorithms were employed to extract high confidence single nucleotide and indel variants for each sample based on mapping and base quality, coverage and variant frequency. Each candidate variant was functionally annotated using tailored scripts based on Ensembl 72 and then screened for presence or absence in several human polymorphisms and population databases such as dbSNP137 [40], 1000 Genomes [41] and ESP6500 [42].

### Genotyping

Restriction fragment-length polymorphism (RFLP) was used to genotype five *LOXL1* SNPs rs16958477, rs1048661, rs3825942, rs2165241 and rs3522, in 200 healthy control subjects, and in 6 XFG patients that were not included in the DNA sequencing of the *LOXL1* gene (for details see Additional file 1). To corraborate that the detection of *LOXL1* SNPs by RFLP was satisfactory, we verified the RFLP technique on 10 DNA samples randomly selected from 99 XFG participants that were initially included in the sequencing of the *LOXL1* gene. In every sample tested we confirmed that DNA sequencing and genotyping by RFLP produced consistent results.

### Statistical analysis

Hardy-Weinberg Equilibrium (HWE) of both groups (cases and controls) was tested using HaploView 4.0 software (Daly Lab, Broad Institute, Cambridge, MA, USA). Comparison of the SNPs allelic frequencies between XFG and control groups was performed using a standard χ^2^ test. Multiple comparisons were corrected by the Bonferroni method. Additionaly we use Sigmaplot v11 software (http://www.sigmaplot.com/) to run a logistic regression analysis in order to control for potential confounders.

The comparison of genotypic frequencies between the XFG and control groups was performed using a χ^2^ test (Pearson correction) with SPSS version 15.0 (IBM Corp., Armonk, NY). Relative risk association was estimated by calculating odds ratios (OR) along with 95 % confidence intervals (CIs) using the methods described in Armitage and Berry [43] and PLINK (v1.07) as described by Purcell et al. [44]. LD plot was generated with HaploView 4.0 software and blocks were defined according to Gabriel et al. algorithm [45]. This software was also used to calculate the haplotype frequencies as well as their association with XFG through a standard χ^2^ test, where *p* < 0.05 was considered statistically significant.

## Results

### *LOXL1* gene sequence analysis

Some demographic characteristics of the subjects of the study are shown in Table 2. Next-generation sequencing of the complete *LOXL1* gene was carried out in 99 of the 105 XFG participants. This task was part of a pilot project aiming to identify rare *LOXL1* gene variants in Spanish subjects with XFG. A preliminar sequencing analysis along the entire *LOXL1* gene identified a total of 212 SNPs, including, rs16958477, rs1048661, rs3825942, rs2165241, and rs3522, subjects of the present study. When the allele frequencies of the 212 SNPs were compared to those reported in the HapMap CEU population study (Utah residents with Northern and Western European ancestry) [46], commonly used as control, significant differences were identified in 49 of them (*p* < 3.42×10^−4^), 66 were not described in the CEU population and 97 showed similar frequencies in both groups (see Additional file 2).

### Association of LOXL1 polymorphisms with XFG

We have determined the allelic frequencies of five *LOXL1* gene variants, SNPs rs16958477, rs1048661, rs3825942, rs2165241, and rs3522 in Spanish XFG cases (*n* = 105) and healthy controls (*n* = 200), as described in Material and Methods. The results are shown in Table 3.

**Table 3:** Allele and genotype frecuencies of five *LOXL1* SNPs in Spanish individuals with XFG and controls

SNP ID		XFG % (*n* = 105)	Control % (*n* = 200)	*p* value	OR (95 % CI)
rs16958477					
Allele	C	57.6	41.2	0.0001	1.94 (1.20–3.13)
	A	42.4	58.8		0.52 (0.32–0.83)
Genotype	CC	32.4	15.5	6.25x10^−4^	2.61 (1.49–4.57)*
	AC	50.5	51.5		0.96 (0.60–1.54)
	AA	17.1	33.0		0.42 (0.23–0.76)
	Total	34/53/18 (CC/CA/AA)	31/103/66 (CC/CA/AA)	4.29x10^−4^	
rs1048661					
Allele	G	84.3	63.7	8.47x10^−8^	3.05 (1.67–5.55)
	T	15.7	36.3		0.22 (0.11–0.43)
Genotype	GG	71.4	40.0	1.80x10^−7^	3.75 (2.25–6.24)*
	GT	25.7	47.0		0.39 (0.23–0.66)
	TT	2.9	13.0		0.42 (0.12–1.52)
	Total	75/27/3 (GG/GT/TT)	80/94/26 (GG/GT/TT)	5.9x10^−7^	
rs3825942					
Allele	G	99.0	84.5	2.54x10^−8^	19.08 (2.57–141.84)
	A	1.0	15.5		0.05 (0.01–0.39)
Genotype	GG	98.1	72.0	3x10^−8^	20.03 (4.78–83.94)*
	GA	1.90	25.0		0.06 (0.02–0.246)
	AA	0	3.0		-
	Total	103/2/0 (GG/GA/AA)	144/50/6 (GG/GA/AA)	2.4x10^−7^	
rs2165241					
Allele	T	80.5	46.50	6.30x10^−16^	4.74 (2.72–8.28)
	C	19.5	53.50		0.21 (0.12–0.37)
Genotype	TT	66.7	20.50	<0.01	7.76 (4.56–13.20)*
	CT	27.6	52.00		0.35 (0.21–0.59)
	CC	5.7	27.50		0.16 (0.06–0.39)
	Total	70/29/6 (TT/TC/CC)	41/104/55 (TT/TC/CC)	<0.01	
rs3522					
Allele	C	61.9	54.0	0.06	0.20 (0.11–0.38)
	T	38.1	46.0		0.53 (0.32–0.85)
Genotype	CC	39.1	30.5	0.13	1.46 (0.89–2.39)*
	CT	45.7	47.0		0.95 (0.59–1.53)
	TT	15.2	22.5		0.62 (0.33–1.16)
	Total	41/48/16 (CC/CT/TT)	61/94/45 (CC/CT/TT)	0.18	

*XFG*; pseudoexfoliation glaucoma, *n*; number of subjects, *OR*; odds ratio, *CI*; confidence interval. The Bonferroni-corrected significance level for the allelic frequencies comparisons was 0.01 (0.05/5). Total indicate the general test of association in the 2- by-3 table of disease-by-genotype. The asterisk (*) indicate the OR values and *p* values derived from comparison of the genotypic frequencies under the recessive model (GG *vs* GT + TT at rs1048661, GG *vs* GA + AA at rs3825942, TT *vs* CT + CC at rs2165241, CC *vs* AC + AA at rs16958477, and CC *vs* CT + TT at rs3522)

Allele frequencies of SNPs rs16958477, rs1048661, rs3825942, and rs2165241, were significantly different when comparing cases and controls and associated with XFG, increasing disease susceptibility from approximately 2- (rs16958477) to 20-fold (rs3825942); however the SNP rs3522 showed similar allele and genotype frequencies in cases and controls (see Table 3). The allele T of the intronic SNP rs2165241 showed the most significant association with XFG (*p* = 6.30x10^−16^; OR = 4.74, 95 % CI: 2.72–8.28). The G allele of SNP rs1048661 (R141L) also showed a strong association with XFG (*p* = 8.47×10^−8^; OR = 3.05, 95 % CI: 1.67–5.55). The G allele of SNP rs3825942 (G153D) was detected in a statistically significant higher frequency in patients with XFG than in controls (*p* = 2.54×10^−8^), with patients being 19-fold more likely to have a G allele than an A allele (OR = 19.08, 95 % CI: 2.57–141.84). Similarly, the C allele of SNP rs16958477, located in the promoter region, showed a significant association with XFG (*p* = 0.0001; OR = 1.94, 95 % CI: 1.20–3.13). All these associations remained significant after the Bonferroni correction for multiple testing (*p* < 0.01). The allelic frequency of the SNP rs3522 in XFG cases was not significantly different from controls (*p* = 0.06).

When the allelic frequencies of the SNPs rs16958477, rs1048661, rs3825942, rs2165241 and rs3522 in the CEU population (*n* = 85) were compared with the Spanish control group (*n* = 200) included in the RFLP study, no significant differences were found. (Table 4).

**Table 4:** Comparison of the allelic frequencies between the study control group and the CEU population

SNP ID	Risk allele	Study Control % (*n* = 200)	CEU population % (*n* = 85)	*p* value
rs16958477	C	41.3	39.4	0.68
rs1048661	G	63.7	63.5	0.96
rs3825942	G	84.5	82.3	0.52
rs2165241	T	46.5	45.9	0.89
rs3522	C	54.0	51.6	0.62

CEU population, Utah residents with Northern and Western European ancestry; n, number of subjects; *p* < 0.05, significant

The observed genotype frequencies of the five *LOXL1* SNPs were in HWE (*p* > 0.01) in both XFG and control groups. Statistically significant differences were observed between XFG subjects and controls when the genotypic frequencies for each of the four SNPs with significantly increased allelic frequency in cases (rs16958477, rs1048661, rs3825942 and rs2165241) were compared (see Table 3).

The frequency of genotype GG at SNP rs3825942 was significantly higher in XFG than in controls under the recessive association model (*p* = 3×10^−8^, GG vs GA + AA) conferring approximately 20-fold increased risk for XFG (OR = 20.03, 95 % CI: 4.78–83.94) whereas the genotypes GA and AA could protect from the disease. The genotype AA was detected only in controls.

Genotype GG of rs1048661 was significantly elevated in XFG, conferring more than 3-fold increased risk for XFG (*p* = 1.8×10^−7^, GG vs GT + TT; OR = 3.75, 95 % CI: 2.25-6.24).

Genotype TT of rs2165241 was also strongly associated with XFG, being found in the 66.67 % of XFG patients and only in 20.5 % of controls (*p* < 0.01, TT vs CT + CC). This genotype confers more than 7-fold increased risk to XFG (OR = 7.76, 95 % CI: 4.56–13.20).

Significant association of the genotype CC of rs16958477 (*p* = 6.25×10^−4^, CC vs AC + AA) was observed with XFG, conferring approximately 2.5-fold increased risk to XFG disease (OR = 2.61, 95 % CI: 1.49–4.57).

In the studied Spanish population, the genotype frequencies of SNP rs3522 did not differ significantly between groups (*p* = 0.13, CC vs CT + TT), suggesting that this SNP is not associated with XFG.

The logistic regression multivariate analysis (multivariate linear regression analysis and backwards stepwise regression analysis) indicated that the covariates sex and age are not predictors of disease in an individual and only rs1048661 and rs3825942 appear to be essential in this model. The five SNPs studied are not completely independent among themselves, since they are part of the same gene, thus the logistic regression analysis detects collinearity among them, providing redundant information in the model.

### Haplotype analysis and linkage disequilibrium

**Fig. 1:**
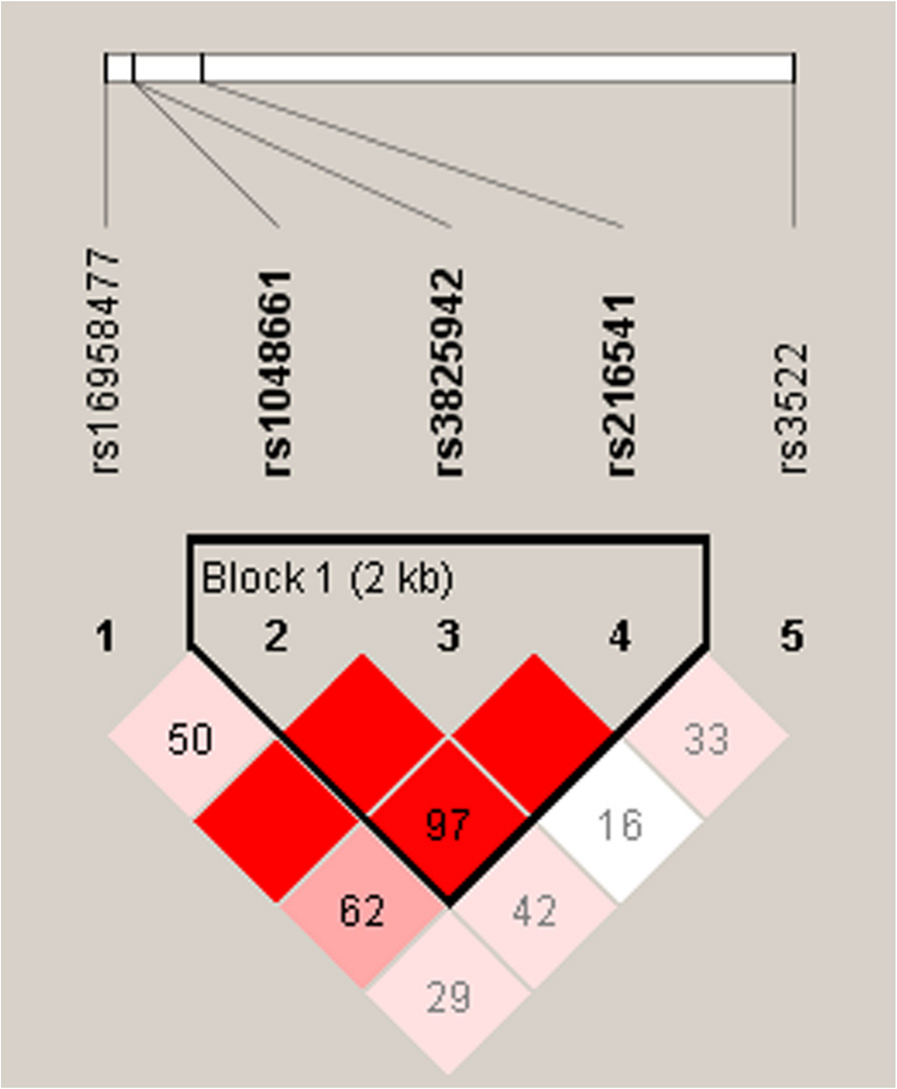
*LOXL1* linkage disequilibrium (LD) plot. LD plot of the SNPs rs16958477, rs1048661, rs3825942, rs2165241 and rs3522 of *LOXL1*. The number in the diamond refers to D’ (100×D’). The LD block was defined according to the standard confidence intervals. The strength of LD is depicted by red intensity, which moves from white to red as D’ progresses from 0 to 100

Pairwise linkage disequilibrium (LD) analysis identified one block (2 kb) consisting of three SNPs (rs1048661, rs3825942 and rs2165241) that were in strong LD, as observed by the D’ value (Fig. 1). This analysis showed that rs3825942 was in complete LD (Coefficient of LD [D’] = 1.00) with rs1048661 and rs2165241. The SNPs rs1048661 and rs2165241 were also in strong LD (D’ = 0.978). Weaker LD was found between pairs rs16958477 and rs2165241, and between rs16958477 and rs1048661 (D’ = 0.62 and 0.5, respectively).

The haplotype analysis identified six of the sixteen theoretically possible haplotypes formed by four SNPs (rs16958477, rs1048661, rs3825942 and rs2165241). The frequency of these haplotypes and their association with XFG are shown in Table 5. The CGGT haplotype, containing all four risk alleles, and the AGGT haplotype, which is composed of the protective allele of rs16958477 and three risk alleles of the other three SNPs, were significantly associated with XFG (*p* = 4.5×10^−6^, and *p* = 8.8×10^−6^), conferring more than 2-fold increased disease risk (OR = 2.21, 95 % CI: 1.36–3.59 for CGGT and OR = 2.53, 95 % CI: 1.41–4.55 for AGGT).

**Table 5:** Haplotype analysis of *LOXL1* SNPs in XFG cases and controls participants in a Spanish population

SNPs alleles				Haplotype frequency		Association test between XFG and Controls	
rs16958477	rs1048661	rs3825942	rs2165241	XFG *n* = 105	Control *n* = 200	*p* value	OR (95% CI)
C	G	G	T	0.52	0.33	4.5×10^-6^	2.21 (1.36–3.59)
A	T	G	C	0.11	0.28	7.1×10^-7^	0.30 (0.15–0.60)
A	G	G	T	0.28	0.14	8.8×10^-6^	2.53 (1.41–4.55)
A	G	A	C	0.005	0.15	9.6×10^-9^	0.03 (0.00–0.43)
C	T	G	C	0.04	0.08	0.08	0.98 (0.41–2.35)
A	G	G	C	0.02	0.01	0.52	1.51 (0.25- 8.96)

*XFG*; pseudoexfoliation glaucoma, *n*; number of subjects. Individual p-values and odds ratios (OR) between exfoliation glaucoma and control are provided for each of the haplotypes compared with all the other haplotypes

In contrast, the haplotypes ATGC and AGAC were found to be protective from XFG in our study (*p* = 7.1×10^−7^; OR = 0.30, 95 % CI: 0.15-0.60 for ATGC and *p* = 9.6×10^−9^; OR = 0.03, 95 % CI: 0.00–0.43 for AGAC). Finally, the AGGC haplotype, despite carrying the risk alleles for the two nonsynonymous coding SNPs, showed no association with XFG (*p* = 0.52). This haplotype appeared in a very low frequency in both groups (2.1 % in XFG and 1.4 % in control). Similarly, no association between the CTGC haplotype and XFG was found (*p* = 0.08).

## Discussion

The *LOXL1* gene is so far the best known genetic risk factor predisposing individuals to XFG. The association of the *LOXL1* SNPs rs1048661, rs3825942, and rs2165241 with XFG has been reported in multiple populations of different ethnic backgrounds and geographic locations around the world. However, there is limited information on the Spanish XFG population. Interestingly, depending on the ethnic group of the individual, specific alleles of these SNPs may or may not confer increased risk of XFS/XFG (Table 1). Since risk-associated allele frequencies are also high in control populations, it has been suggested that other genes and/or others *LOXL1* variants may contribute to the risk of developing XFG. Thus, sequencing of the entire *LOXL1* gene, including intronic, exonic, promoter and regulatory regions, in XFG cases from different populations could contribute to identify such variants.

As an initial step to determine the role of *LOXLI* gene variants in Spanish XFG patiens, we compared the frequency of identified variants with those available from the CEU population, which is included in the Haplotype Mapping Project (HapMap), and commonly used as control in association studies. Although new *LOXL1* gene variants were identified when compared to the CEU population, we can not rule out any false negative result. Future sequencing of the *LOXL1* gene in the Spanish control group will provide a full scope on the frequency of all the variants. As a result of this analysis we identified no other *LOXL1* gene coding variants associated with XFG, apart from those previously described. Thus, in the present case–control association study, we selected five known SNPs variants, i.e., the three *LOXL1* gene most common sequence variants (rs2165241T_C, rs1048661G_T, and rs3825942G _A) and others two (rs16958477A_C and rs3522C_T) to test them in a bigger Spanish population. Although the association of the latter two variants with XFG did not survive correction for multiple testing (*p* = 0.0007 and *p* = 0.03, respectively) in our pilot study, these SNPs were included in our subsequent analysis because they had been previously related with the disease in another glaucomatous populations. In contrast, the recently described coding variant rs41435250, located in exon 1 and identified as a risk variant in South Indian and Mexican subjects [32, 37], has not been included in this analysis. This SNP did not associate with XFG in our pilot study (*p* = 0.4), which is in agreement with the result obtained in other populations including South African subjects (*p* = 0.49) [34] and a Saudi Arabian population, where it was found to be monoallelic [36]. Moreover, we also identified *LOXL1* variants no previously reported (see Additional file 2). Additional risk-associated alleles of these unknown variants with XFG remains to be studied in future experiments.

Evidence from many populations support the assertion that *LOXL1* SNPs are strongly associated with XFS/XFG, while in other populations, the same alleles have a protective effect. This suggests that variants could be markers for the disease rather than having a functional role. Our results are consistent with a strong genetic association between three of the most common SNPs of *LOXL1* (rs1048661, rs3825942, rs2165241) with XFG in the Spanish population. These results are generally in good agreement with findings reported in other European XFG populations including those from Iceland, Sweden, Finland, Germany and Italy [20, 47, 48], white North American XFG populations [49–51] and a population from South India [32]. However, our results are in contrast with the lack of relationship between XFG and the G allele of rs1048661 in other Spanish population [35]. Furthermore, we also found differences in the association of other *LOXL1* SNPs (i.e. rs16958477 and rs3522) with XFG, between the Spanish and other populations.

Within the promoter region of *LOXL1*, the SNP rs16958477 has been described as a transcriptionally relevant SNP, and it has been shown to be a risk-associated allele with XFG in a caucasian North American population. While the A allele of rs16958477 was identified as a XFG risk factor in the caucasian USA population [38], our results showed that in the Spanish population the C allele conferred the highest risk (see Table 3). This risk allele has also been observed in a South Indian population [32]. Our studies are also in contrast with the lack of association of the A or C variants of rs16958477 with XFG in the black South African population [34].

Our results also suggested that there was no significant association between the C allele of rs3522 and XFG in the Spanish population. This is in agrement with earlier observations reported in North American [49], Saudi Arabian [36] and South Indian [32] populations. Although this variant was found to be associated with XFG (*p* = 0.02) in a black South African population, it did not survive correction for multiple testing in that population [34].

To further emphasize the main differences among *LOXL1* alleles, conferring or not a risk to XFG in different populations, a comparative analysis of the 3 main risk alleles analyzed in the Spanish population with XFG and populations with XFS/XFG around the world is summarized in Table 1. In this analysis, the observed genetic differences between Asian populations and Scandinavian, Western Europe or Spanish populations are striking. For example, in the Japanese populations, the T allele of rs1048661 and the C allele of rs2165241 conferred risk of XFG, respectively, whereas in European population, the G allele of rs1048661 and the T allele of rs2165241 are more closely associated with XFG. Another significant difference is the association between the A allele of rs3825942 and XFG in black South Africans, whereas the G allele is the risk-allele in all other populations studied so far. These results highlight the differences in XFG risk in different populations around the world.

The Spanish population studied here comprised of approximately 75 and 66 % of XFG and control participants from the Northwestern regions of Spain, where the prevalence of XFG is higher than in other regions of Spain, which could reflect a different genetic background of the subjects from these regions. Thus, differences in the geographic origin of the Spanish populations could explain the discrepancies between our results and those reported recently in a different Spanish population [35]. It is well known that the frequencies of risk alleles and their association with XFG vary not only among different ethnic groups, but also among populations of the same country, as described in studies with differents populations from Greek, China or India (Table 1).

Our study corroborate that under the recessive model of inheritance, the genotype TT of the intronic SNP rs2165241 showed the strongest association with XFG, with a 7.7-fold disease risk increase; additionally, the genotypes GG of rs1048661, GG of rs3825942, and CC of rs16958477 were also significantly associated with XFG (Table 3).

Another valuable piece of evidence supporting the identification of high risk-associated alleles in the Spanish population with XFS/XFG, is the haplotype association analysis. The haplotype analysis using four SNPs, which were strongly associated with XFG in the Spanish population (rs16958477, rs1048661, rs3825942, and rs2165241), identified six different haplotypes, including CGGT and AGGT which showed increased risk (see Table 5). These findings are also in agreement with previous observations in other caucasians populations [47, 50–53]. In contrast, ATGC and AGAC haplotypes were relatively infrequent among XFG cases in the Spanish population, compared to controls, suggesting that they likely do not confer risk or predispose patients to the disease. Although the two identified risk haplotypes (CGGT and AGGT) are present in 80 % of the XFG cases, increasing the risk of XFG more than 4-fold relative to the rest of the haplotypes (ATGC, AGAC, CTGC and AGGC) and more than 55-fold relative to the low-risk (AGAC) haplotype, they were also detected in control subjects (46 %). The high prevalence of *LOXL1* risk alleles/haplotypes has been reported in all populations examined to date. This, in addition to the fact that the disease-associated haplotype differs between different ethnicities, could suggest that although these variants are not disease-causing, they could be in LD with other *LOXL1* variants responsible for disease.

In light of these results, the T allele of the intronic SNP rs2165241 is likely the main genetic risk-associated allele with XFG in the Spanish population. The risk conferred by the G allele of rs1048661 and rs3825942 could be due to their linkage disequilibrium with rs2165241. However, their association with other variant(s) (not included in this analysis) should not be ruled out. Although *LOXL1* variants remain the main risk factor of XFG, increasing evidence suggests that additional genes and/or environmental factors are likely involved in the development of XFG [54, 55]. Recently, Wiggs et al. showed that *Loxl1* null mice did not exhibit ocular nor systemic features of XFS and the absence of LOXL1protein did not result in deposition of exfoliative material or glaucoma. An alteration in the structure and/or function of the LOXL1 protein, rather than its lack, could be related to the deposition of exfoliative material [56].

## Conclusions

We studied the association of *LOXL1* gene variants rs16958477, rs1048661, rs3825942, rs2165241 and rs3522 with XFG in patients from the Northern region of Spain, where XFS is highly prevalent among the general population. The allelic and genotypic frequencies of the SNPs studied suggested that the alleles G of rs1048661 and rs3825942, and the allele T of rs2165241 are likely the main genetic risk variants associated with XFG in the Spanish population. This information adds new support to the distinct risk association frequencies of *LOXL1* alleles with XFG in Western European and Asian populations, possibly underlying ethnicity and demographic differences. Finally, sequencing of the *LOXL1* gene from additional XFG and control cases may provide means to identify and test additional rare variants with potential functional roles in the pathogenesis of this disease.

## Additional files

Additional file 1:
**Next generation sequencing and PCR-RFLP genotyping details.** Details on the amplification and sequencing of the 39.2-Kb DNA region containing the complete *LOXL1* gene, and 5´ and 3´ flanking untranslated (UT) are provided. The process of PCR_RFLP genotyping of the *LOXL1* SNPs rs16958477, rs1048661, rs3825942, rs2165241 and rs3522 is described. (DOCX 20 kb) Additional file 2:
**List of variants identified by sequencing of the** ***LOXL1*** **gene in a Spanish population with XFG.** The location, nucleotide position and risk allele for each SNP are indicated. Comparison of the allele frequencies of each SNP in the Spanish and CEU population (HapMap Project) used as control, are shown. The SNP identification (SNP ID) depicted by “ND” indicates it has not been previously described. When the allele frequency for a specific SNP in the CEU population is depicted by a dot (.) indicates that it has not been described in this population. The asterisk indicates that nucleotides are numbered using as reference GenBank accession number NC_000015.9. *n* = number of subjects. The Bonferroni-corrected significance level was 3.42×10^−4^ (0.05/146). Significant variants are listed in bold (DOCX 207 kb)

## References

[1] Naumann GO, Schlötzer-Schrehardt U, Küchle M (1998). Pseudoexfoliation syndrome for the comprehensive ophthalmologist. Intraocular and systemic manifestations. Ophthalmology.

[2] Schlötzer-Schrehardt U, Naumann GOH (2006). Ocular and systemic pseudoexfoliation syndrome. Am J Ophthalmol.

[3] Ritch R, Schlötzer-Schrehardt U (2001). Exfoliation syndrome. Surv Ophthalmol.

[4] Ringvold A (1999). Epidemiology of the pseudo-exfoliation syndrome. Acta Ophthalmol Scand.

[5] Miyazaki M, Kubota T, Kubo M, Kiyohara Y, Iida M, Nose Y, Ishibashi T (2005). The prevalence of pseudoexfoliation syndrome in a Japanese population: the Hisayama study. J Glaucoma.

[6] Mitchell P, Wang JJ, Hourihan F (1999). The relationship between glaucoma and pseudoexfoliation: the Blue Mountains Eye Study. Arch Ophthalmol.

[7] Cashwell LF, Shields MB (1988). Exfoliation syndrome. Prevalence in a southeastern United States population. Arch Ophthalmol.

[8] Foster PJ, Seah SK (2005). The prevalence of pseudoexfoliation syndrome in Chinese people: the Tanjong Pagar Survey. Br J Ophthalmol.

[9] Young AL, Tang WW, Lam DS (2004). The prevalence of pseudoexfoliation syndrome in Chinese people. Br J Ophthalmol.

[10] Challa P (2009). Genetics of pseudoexfoliation syndrome. Curr Opin Ophthalmol.

[11] Lantukh VV, Piatin MM (1982). Features of ocular pathology among the indigenous inhabitants of Chukotka. Vestn Oftalmol.

[12] Moreno Montañés J, Alcolea Paredes A, Campos García S (1989). Prevalence of pseudoexfoliation syndrome in the northwest of Spain. Acta Ophthalmol (Cophen).

[13] Schlötzer-Schrehardt U, Naumann GOH (1995). Trabecular meshwork in pseudoexfoliation syndrome with and without open-angle glaucoma. A morphometric, ultrastructural study. Invest Ophthalmol Vis Sci.

[14] Jeng SM, Karger RA, Hodge DO, Burke JP, Johnson DH, Good MS (2007). The risk of glaucoma in pseudoexfoliation syndrome. J Glaucoma.

[15] Ritch R, Schlötzer-Schrehardt U, Konstas AG (2003). Why is glaucoma associated with exfoliation syndrome?. Prog Retin Eye Res.

[16] Ritch R (2008). Exfoliation syndrome: beyond glaucoma. Arch Ophthalmol.

[17] Forsius H, Forsman E, Fellman J, Eriksson AW (2002). Exfoliation syndrome: frequency, gender distribution and association with climatically induced alterations of the cornea and conjunctiva. Acta Ophthalmol Scand.

[18] Damji KF, Bains HS, Stefansson E, Loftsdottir M, Sverrisson T, Thorgeirsson E, Jonasson F, Gottfredsdottir M, Allingham RR (1998). Is pseudoexfoliation syndrome inherited? A review of genetic and nongenetic factors and a new observation. Ophthalmic Genet.

[19] Orr AC, Robitaille JM, Price PA, John R, Hamilton JR, Falvey DM, De Saint-Sardos AG, Pastermak S, Guemsey DL (2001). Exfoliation syndrome: clinical and genetic features. Ophthalmic Genet.

[20] Thorleifsson G, Magnusson KP, Sulem P, Walters GB, Gudbjartsson DF, Stefansson H, Jonsson T, Jonasdottir A, Stefansdottir G, Masson G (2007). Common sequence variants in the LOXL1 gene confer susceptibility to exfoliation glaucoma. Science.

[21] Chen L, Jia L, Wang N, Tang G, Zhang C, Fan S, Liu W, Meng H, Zeng W, Liu N, Wang H, Jia H (2009). Evaluation of LOXL1 polymorphisms in exfoliation syndrome in a Chinese population. Mol Vis.

[22] Fuse N, Miyazawa A, Nakazawa T, Mengkegale M, Otomo T, Nishida K (2008). Evaluation of LOXL1 polymorphisms in eyes with exfoliation glaucoma in Japanese. Mol Vis.

[23] Hayashi H, Gotoh N, Ueda Y, Nakanishi H, Yoshimura N (2008). Lysyl oxidase-like 1 polymorphisms and exfoliation syndrome in the Japanese population. Am J Ophthalmol.

[24] Mabuchi F, Sakurada Y, Kashiwagi K, Yamagata Z, Iijima H, Tsukahara S (2008). Lysyl oxidase-like 1 gene polymorphisms in Japanese patients with primary open angle glaucoma and exfoliation syndrome. Mol Vis.

[25] Mori K, Imai K, Matsuda A, Ikeda Y, Naruse S, Hitora-Takeshita H, Nakano M, Taniguchi T, Omi N, Tashiro K, Kinoshita S (2008). LOXL1 genetic polymorphisms are associated with exfoliation glaucoma in the Japanese population. Mol Vis.

[26] Ozaki M, Lee KY, Vithana EN, Yong VH, Thalamuthu A, Mizoguchi T, Venkatraman A, Aung T (2008). Association of LOXL1 gene polymorphisms with pseudoexfoliation in the Japanese. Invest Ophthalmol Vis Sci.

[27] Tanito M, Minami M, Akahori M, Kaidzu S, Takai Y, Ohira A, Iwata T (2008). LOXL1 variants in elderly Japanese patients with exfoliation syndrome/glaucoma, primary open-angle glaucoma, normal tension glaucoma, and cataract. Mol Vis.

[28] Park do Y, Won HH, Cho HK, Kee C (2013). Evaluation of lysyl oxidase-like 1 gene polymorphisms in pseudoexfoliation syndrome in a Korean population. Mol Vis.

[29] Sagong M, Gu BY, Cha SC (2011). Association of lysyl oxidase-like 1 gene polymorphisms with exfoliation syndrome in Koreans. Mol Vis.

[30] Lee KY, Ho SL, Thalamuthu A, Venkatraman A, Venkataraman D, Pek DC, Aung T, Vithana EN (2009). Association of LOXL1 polymorphisms with pseudoexfoliation in the Chinese. Mol Vis.

[31] Ramprasad VL, George R, Soumittra N, Sharmila F, Vijaya L, Kumaramanickavel G (2008). Association of non-synonymous single nucleotide polymorphisms in the LOXL1 gene with pseudoexfoliation syndrome in India. Mol Vis.

[32] Dubey SK, Hejtmancik JF, Krishnadas SR, Sharmila R, Haripriya A, Sundaresan P (2014). Lysyl Oxidase-Like 1 Gene in the Reversal of Promoter Risk Allele in Pseudoexfoliation Syndrome. JAMA Ophthalmol.

[33] Rautenbach RM, Bardien S, Harvey J, Ziskind A (2011). An investigation into LOXL1 variants in black South African individuals with exfoliation syndrome. Arch Ophthalmol.

[34] Williams SE, Whigham BT, Liu Y, Carmichael TR, Qin X, Schmidt S, Ramsay M, Hauser MA, Allingham RR (2010). Major LOXL1 risk allele is reversed in exfoliation glaucoma in a black South African population. Mol Vis.

[35] de Juan-Marcos L, Escudero-Domínguez FA, Hernández-Galilea E, Cabrillo-Estévez L, Cruz-González F, Cieza-Borrella C, Sánchez-Barba M, González-Sarmiento R (2014). Association of lysyl oxidase-like 1 gene polymorphisms in pseudoexfoliation syndrome and pseudoexfoliation glaucoma in a Spanish population. Ophthalmic Genet.

[36] Abu-Amero KK, Osman EA, Dewedar AS, Schmidt S, Allingham RR, Al-Obeidan SA (2010). Analysis of LOXL1 polymorphisms in a Saudi Arabian population with pseudoexfoliation glaucoma. Mol Vis.

[37] Guadarrama-Vallejo D, Miranda-Duarte A, Zenteno JC (2013). The T allele of lysyl oxidase-like 1 rs41435250 is a novel risk factor for pseudoexfoliation syndrome and pseudoexfoliation glaucoma independently and through intragenic epistatic interaction. Mol Vis.

[38] Fan BJ, Pasquale LR, Rhee D, Li T, Haines JL, Wiggs JL (2011). LOXL1 promoter haplotypes are associated with exfoliation syndrome in a US Caucasian population. Invest Ophthalmol Vis Sci.

[39] Ferrell G, Lu M, Stoddard P, Sammel MD, Romero R, Strauss JF, Matthews CA (2009). A single nucleotide polymorphism in the promoter of the LOXL1 gene and its relationship to pelvic organ prolapse and preterm premature rupture of membranes. Reprod Sci.

[40] The single nucleotide Polymorphism 137 (dbSNP137) Database [http://www.ncbi.nlm.nih.gov/snp/]

[41] 1000 Genomes Database [http://www.1000genomes.org/]

[42] Exome Sequencing Project 6500 (ESP6500) Database [http://evs.gs.washington.edu/EVS/]

[43] Armitage P, Berry G. Statistical methods in medical research. 3rd ed. Oxford, Editor; Boston: Blackwell Scientific Publications; 1994.

[44] Purcell S, Neale B, Todd-Brown K, Thomas L, Ferreira MA, Bender D, Maller J, Sklar P, de Bakker PI, Daly MJ, Sham PC (2007). PLINK: a tool set for whole-genome association and population-based linkage analyses. Am J Hum Genet.

[45] Gabriel SB, Schaffner SF, Nguyen H, Moore JM, Roy J, Blumenstiel B, Higgins J, DeFelice M, Lochner A, Faggart M, Liu-Cordero SN, Rotimi C, Adeyemo A, Cooper R, Ward R, Lander ES, Daly MJ, Altshuler D (2002). The structure of haplotype blocks in the human genome. Science.

[46] Lundmark PE, Liljedahl U, Boomsma DI, Mannila H, Martin NG, Palotie A, Peltonen L, Perola M, Spector TD, Syvänen AC (2008). Evaluation of HapMap data in six populations of European descent. Eur J Hum Genet.

[47] Lemmelä S, Forsman E, Onkamo P, Nurmi H, Laivuori H, Kivelä T, Puska P, Heger M, Eriksson A, Forsius H, Järvelä I (2009). Association of LOXL1 gene with Finnish exfoliation syndrome patients. J Hum Genet.

[48] Pasutto F, Krumbiegel M, Mardin CY, Paoli D, Lammer R, Weber BH, Kruse FE, Schlotzer-Schrehardt U, Reis A (2008). Association of LOXL1 common sequence variants in German and Italian patients with pseudoexfoliation syndrome and pseudoexfoliation glaucoma. Invest Ophthalmol Vis Sci.

[49] Challa P, Schmidt S, Liu Y, Qin X, Vann RR, Gonzalez P, Allingham RR, Hauser MA (2008). Analysis of LOXL1 polymorphisms in a United States population with pseudoexfoliation glaucoma. Mol Vis.

[50] Fan BJ, Pasquale L, Grosskreutz CL, Rhee D, Chen T, DeAngelis MM, Kim I, del Bono E, Miller JW, Li T, Haines JL, Wiggs JL (2008). DNA sequence variants in the LOXL1 gene are associated with pseudoexfoliation glaucoma in a U.S. clinicbased population with broad ethnic diversity. BMC Med Genet.

[51] Aragon-Martin JA, Ritch R, Liebmann J, O’Brien C, Blaaow K, Mercieca F, Spiteri A, Cobb CJ, Damji KF, Tarkkanen A, Rezaie T, Child AH, Sarfarazi M (2008). Evaluation of LOXL1 gene polymorphisms in exfoliation syndrome and exfoliation glaucoma. Mol Vis.

[52] Metaxaki I, Constantoulakis P, Papadimitropoulos M, Filiou E, Georgopoulos G, Chamchougia A, Papakonstantinou D, Markomichelakis N, Koutsandrea C, Halkiadakis I (2013). Association of lysyl oxidase-like 1 gene common sequence variants in Greek patients with pseudoexfoliation syndrome and pseudoexfoliation glaucoma. Mol Vis.

[53] Malukiewicz G, Lesiewska-Junk H, Linkowska K, Mielnik M, Grzybowski T, Sulima N (2011). Analysis of LOXL1 single nucleotide polymorphisms in Polish population with pseudoexfoliation syndrome. Acta Ophthalmol (Copenh).

[54] Stein JD, Pasquale LR, Talwar N, Kim DS, Reed DM, Nan B, Kang JH, Wiggs JL, Richards JE (2011). Geographic and climatic factors associated with exfoliation syndrome. Arch Ophthalmol.

[55] Pasquale LR, Wiggs JL, Willett WC, Kang JH (2012). The Relationship between caffeine and coffee consumption and exfoliation glaucoma or glaucoma suspect: a prospective study in two cohorts. Invest Ophthalmol Vis Sci.

[56] Wiggs JL, Pawlyk B, Connolly E, Adamian M, Miller JW, Pasquale LR, Haddadin RI, Grosskreutz CL, Rhee DJ, Li T (2014). Disruption of the blood-aqueous barrier and lens abnormalities in mice lacking Lysyl Oxidase-like 1 (LOXL1). Invest Ophthalmol Vis Sci.

[57] Mossböck G, Renner W, Faschinger C, Schmut O, Wedrich A, Weger M (2008). Lysyl oxidase-like protein 1 (LOXL1) gene polymorphisms and exfoliation glaucoma in a Central European population. Mol Vis.

[58] Chiras D, Tzika K, Kokotas H, Oliveira SC, Grigoriadou M, Kastania A, Dima K, Stefaniotou M, Aspiotis M, Petersen MB, Kroupis C, Kitsos G (2013). Development of novel LOXL1 genotyping method and evaluation of LOXL1, APOE and MTHFR polymorphisms in exfoliation syndrome/glaucoma in a Greek population. Mol Vis.

[59] Anastasopoulos E, Coleman AL, Wilson MR, Sinsheimer JS, Yu F, Katafigiotis S, Founti P, Salonikiou A, Theofanis Pappas T, Koskosas A, Katopodi T, Lambropoulos A, Topouzis F (2014). Association of LOXL1 polymorphisms with pseudoexfoliation, glaucoma, intraocular pressure, and systemic diseases in a greek population. The Thessaloniki Eye Study. Invest Ophthalmol Vis Sci.

[60] Yang X, Zabriskie NA, Hau VS, Chen H, Tong Z, Gibbs D, Farhi P, Katz BJ, Luo L, Pearson E, Goldsmith J, Ma X, Kaminoh Y, Chen Y, Yu B, Zeng J, Zhang K, Yang Z (2008). Genetic association of LOXL1 gene variants and exfoliation glaucoma in a Utah cohort. Cell Cycle.

[61] Hewitt AW, Sharma S, Burdon KP, Wang JJ, Baird PN, Dimasi DP, Mackey DA, Mitchell P, Craig JE (2008). Ancestral LOXL1 variants are associated with pseudoexfoliation in Caucasian Australians but with markedly lower penetrance than in Nordic people. Hum Mol Genet.

[62] Kasım B, İrkeç M, Alikaşifoğlu M, Orhan M, Mocan MC, Aktaş D (2013). Association of LOXL1 gene polymorphisms with exfoliation syndrome/glaucoma and primary open angle glaucoma in a Turkish population. Mol Vis.

[63] Micheal S, Khan MI, Akhtar F, Ali M, Ahmed A, den Hollander AI, Qamar R (2012). Role of Lysyl oxidase-like 1 gene polymorphisms in Pakistani patients with pseudoexfoliative glaucoma. Mol Vis.

[64] Jaimes M, Rivera-Parra D, Miranda-Duarte A, Valdés G, Zenteno JC (2012). Prevalence of high-risk alleles in the LOXL1 gene and its association with pseudoexfoliation syndrome and exfoliation glaucoma in a Latin American population. Ophthalmic Genet.

[65] Mayinu, Chen X (2011). Evaluation of LOXL1 polymorphisms in exfoliation syndrome in the Uygur population. Mol Vis.

