# Supplementary material for: LOXL1 gene variants and their association with pseudoexfoliation glaucoma (XFG) in Spanish patients
Source: BMC Med Genet. 2015 Aug 31;16:72. doi: 10.1186/s12881-015-0221-y (PMC4593192; doi:10.1186/s12881-015-0221-y)
Supplement: Additional file 1: — Next generation sequencing and PCR-RFLP genotyping details. Details on the amplification and sequencing of the 39.2-Kb DNA region containing the complete LOXL1 gene, and 5´ and 3´ flanking untranslated (UT) are provided. The process of PCR_RFLP genotyping of the LOXL1 SNPs rs16958477, rs1048661, rs3825942, rs2165241 and rs3522 is described. (DOCX 20 kb) [file 12881_2015_221_MOESM1_ESM.docx]

**Additional file 1. Next generation sequencing and PCR-RFLP genotyping details.**

**Next generating sequencing**

***LOXL1* gene amplification:** The commercial sets of primers used to amplify the 39.2-Kb DNA region, containing the complete *LOXL1* gene (25,690 bp) and the 5´ (7370 bp) and 3´ (6170 bp) flanking untranslated (UT) regions, were the following: Hs_LOXL1_01a_LR for fragment 1 amplification (10464bp; Chr15: 74211419-74221883), Hs_LOXL1_02a_LR for fragment 2 amplification (10800bp; Chr15: 74221523-74232323), Hs_LOXL1_03a_LR for fragment 3 amplification (9965bp; Chr15: 74231398-74241363) and Hs_LOXL1_04a_LR for fragment 4 amplification (9993bp; Chr15: 74240655-74250648) (Applied Biosystems, Langen, Germany).

Amplification of fragments 1, 2 and 3 were run with a four-stage program with an increased extension time in the third and fourth stages: 93 °C for 3 min; 93 °C for 15 s, 62 °C for 30 s, 68 °C for 10 min over 10 cycles; 93 °C for 15 sec, 62 °C for 30 sec, 68 °C for 10 min over 15 cycles in which extension time is increased by increments of 20 sec in each cycle; 93 °C for 15 sec, 64 °C for 30 sec, 68 °C for 15 min over 10 cycles in which extension time is increased by increments of 20 sec in each cycle. Addition of 7.5% DMSO was required for amplification of fragment 1. The PCR program for fragment 4 was a three-stage program with an increased extension time in stage three: 93 °C for 3 min; 93 °C for 15 sec, 62 °C for 30 sec, 68 °C for 10 min over 10 cycles; 93 °C for 15 sec, 62 °C for 30 sec, 68 °C for 10 min over 25 cycles in which extension time is increased by increments of 20 sec in each cycle.

**Sequencing**: Amplicons were purified and the libraries were prepared as described in the main text of the manuscript. Then the libraries were sequenced using the platform HiScanSQ under the following settings: i) Run type: Single Read, Multiplexing, Number of cycles: Read 1=50 nt targeted sequencing, Read 2=7 nt index sequencing; ii) Flowcell Version: HiSeq Flow Cell v3; iii) Flow cells were clustered using TruSeq SR Cluster Kit v3 - cBot – HS; iv) the sequencing chemistry used was TruSeq SBS Kit v3 - HS (50-cycles), with Illumina software RTA Version 1.13.48 and CASAVA Version 1.8.1.

**PCR-RFLP Genotyping**

Restriction fragment-length polymorphism (RFLP) was used to genotype the *LOXL1* SNPs rs16958477, rs1048661, rs3825942, rs2165241 and rs3522.

Genotyping of the two nonsynonymous exonic SNPs, rs1048661 and rs3825942, was performed by digesting PCR generated amplicons (432 bp long) containing these SNPs with the enzymes *SmaI* and *HinfI* respectively. In the case of the SNP rs1048661, PCR fragments with the risk allele G at codon 141 (141R allele) yielded three fragments of 201 bp, 146 bp and 85 bp long each, upon digestion with *SmaI*. In contrast, fragments with the allele T at codon 141 (141L allele) lacked the *SmaI* restriction site, yielding only 2 fragments of 347 bp and 85 bp long. However, PCR amplicons with the heterozygous genotype GT yielded all the ~~4~~ fragments (347 bp, 201 bp, 146 bp and 85 bp) upon digestion with *SmaI*.

Genotyping of the SNP rs3825942 was carried out by generating amplicons of 432 bp long containing the SNP, and *Hinf I* restriction enzyme digestion. Amplicons with A at codon 153 (153D allele) were sensitive to *Hinf I* digestion resulting in 2 fragments of 322 bp and 110 bp, whereas amplicons with G at codon 153 (153G allele) lacked the *Hinf I* restriction site and therefore were resistant to digestion, resulting in an intact fragment of 432 bp. Amplicons with the heterozygous GA genotype yielded 3 DNA fragments (432 bp, 322 bp, and 110 bp) upon digestion with *Hinf I*.

Genotyping of intronic SNP rs2165241 was performed by digesting a 536 bp PCR amplicon containing this SNP with the enzyme *SspI*. The presence of the risk allele T resulted in two fragments of 331 bp and 205 bp, whereas the presence of the allele C caused resistance to *SspI* digestion. The presence of both alleles (TC) produced three fragments of 536 bp, 331 bp and 205 bp, respectively.

Amplicons harboring the SNP rs16958477 were genotyped using the restriction enzyme *BauI.* PCR-generated amplicons 416 bp long containing the risk allele C were sensitive to *BauI* digestion, creating two fragments of 246 bp and 170 bp fragments, whereas, amplicons with the allele A were resistant to *BauI* digestion. Amplicons containing the heterozygous AC produced three fragments (416 bp, 246 bp and 170 bp), upon digestion with *BauI.*

Finally, genotyping of the SNP rs3522 was carried out by digesting 309-bp PCR-generated amplicons containing the SNP with the enzyme *BsaHI*. The presence of the allele C in the SNP conferred sensitivity to *BsaHI* digestion, yielding two fragments of 204 bp and 105 bp. DNA amplicons containing the allele T at the same position resisted *BsaHI* digestion, resulting an intact undigested fragment of 309 bp. Digestion of amplicons containing the heterozygous CT genotype with *BsaHI* yielded three fragments (i.e., 309 bp, 204 bp and 105 bp).
