# Supplementary material for: LOXL1 gene variants and their association with pseudoexfoliation glaucoma (XFG) in Spanish patients
Source: BMC Med Genet. 2015 Aug 31;16:72. doi: 10.1186/s12881-015-0221-y (PMC4593192; doi:10.1186/s12881-015-0221-y)
Supplement: Additional file 2: — List of variants identified by sequencing of the LOXL1 gene in a Spanish population with XFG. The location, nucleotide position and risk allele for each SNP are indicated. Comparison of the allele frequencies of each SNP in the Spanish and CEU population (HapMap Project) used as control, are shown. The SNP identification (SNP ID) depicted by “ND” indicates it has not been previously described. When the allele frequency for a specific SNP in the CEU population is depicted by a dot (.) indicates that it has not been described in this population. The asterisk indicates that nucleotides are numbered using as reference GenBank accession number NC_000015.9. n = number of subjects. The Bonferroni-corrected significance level was 3.42×10^−4^ (0.05/146). Significant variants are listed in bold (DOCX 207 kb) [file 12881_2015_221_MOESM2_ESM.docx]

**Additional file 2**

**List of variants identified by sequencing of the *LOXL1* gene in a Spanish population with XFG.**

The location, nucleotide position and risk allele for each SNP is indicated. Comparison of the allele frequencies of each SNP in the Spanish and CEU population (HapMap Project), used as control, are shown. The SNP identification (SNP ID) depicted by ”ND” indicates it has not been previously described. When the allele frequency for a specifc SNP in the CEU population is depicted by a dot (.), indicates that it has not been described in this population.

The asterisk indicates that nucleotides are numbered as in GenBank accession number NC_000015.9. XFG, pseudoexfoliation glaucoma, n = number of subjects. The Bonferroni-corrected significance level was 3.42x10^-4^ (0.05/146). In bold significant variants.

| **Location** | **Nucleotide position*** | **SNP ID** | **Risk Allele** | **Spanish population**  **XFG**  **Allele Frequencies (n=99)** | **CEU population**  **Control**  **Allele**  **Frequencies (n=85)** | **p value** |
| --- | --- | --- | --- | --- | --- | --- |
| Promotor | g.74211548C>T | rs4461027 | C | 0.54 | 0.39 | 0.0068 |
| Promotor | g.74211717T>C | rs7177530 | T | 0.54 | 0.39 | 0.0068 |
| Promotor | 74211754 | rs116974080 | G | 0.99 | 0.94 | 0.01 |
| Promotor | 74212472 | ND | A | 0.005 | . | . |
| Promotor | 74212952 | rs56062631 | T | 0.99 | 0.94 | 0.01 |
| Promotor | 74213151 | rs56116666 | G | 0.99 | 0.94 | 0.01 |
| Promotor | 74213172 | ND | A | 0.005 | . | . |
| Promotor | 74213253 | ND | C | 0.02 | . | . |
| Promotor | 74213357 | rs28542042 | C | 0.79 | 0.65 | 0.0032 |
| Promotor | 74213872 | rs7167367 | A | 0.55 | 0.51 | 0.40 |
| Promotor | 74214506 | rs76148715 | A | 0.99 | 0.94 | 0.01 |
| Promotor | g.74214554C>T | rs76913573 | C | 0.99 | 0.94 | 0.01 |
| Promotor | g.74214883A>G | rs7173684 | A | 0.50 | 0.38 | 0.03 |
| Promotor | 74215086 | rs7174531 | T | 0.005 | . | . |
| Promotor | 74215292 | ND | A | 0.005 | . | . |
| Promotor | 74215318 | rs148381143 | T | 0.005 | . | . |
| Promotor | 74215551 | rs4886761 | T | 0.48 | 0.38 | 0.06 |
| Promotor | 74215820 | ND | A | 0.005 | . | . |
| Promotor | 74216142 | ND | T | 0.005 | . | . |
| Promotor | 74216605 | rs12914565 | C | 0.73 | 0.64 | 0.07 |
| Promotor | 74216759 | rs8026259 | C | 0.27 | 0.17 | 0.02 |
| Promotor | 74216765 | rs192275913 | T | 0.02 | . | . |
| Promotor | 74217071 | rs137907924 | G | 0.99 | . | . |
| Promotor | 74217126 | ND | A | 0.005 | . | . |
| Promotor | 74217222 | rs142446785 | A | 0.02 | 0.006 | 0.24 |
| Promotor | 74217224 | rs76712224 | A | 0.005 | . | . |
| Promotor | 74217733 | ND | A | 0.005 | . | . |
| Promotor | 74217839 | rs6495084 | T | 0.94 | 0.97 | 0.20 |
| Promotor | 74218178 | rs12708513 | C | 0.94 | 0.97 | 0.20 |
| Promotor | g.74218466A>C | rs16958477 | C | 0.57 | 0.39 | 0.0007 |
| Promotor | 74218671 | ND | T | 0.005 | . | . |
| Exon 1 | 74218896 | ND | A | 0.005 | . | . |
| Exon 1 | 74219163 | ND | A | 0.005 | . | . |
| **Exon 1** | **g.74219546G>T** | **rs1048661** | **G** | **0.85** | **0.64** | **<3.42x10^-4^** |
| **Exon 1** | **g.74219582G>A** | **rs3825942** | **G** | **0.99** | **0.83** | **<3.42x10^-4^** |
| Exon 1 | 74220000 | rs41429348 | T | 0.02 | 0.05 | 0.07 |
| Exon 1 | 74220084 | rs41435250 | T | 0.02 | 0.006 | 0.40 |
| Intron 1 | 74220404 | rs73446383 | C | 0.02 | . | . |
| Intron 1 | 74220514 | rs189207294 | A | 0.005 | . | . |
| Intron 1 | 74220591 | ND | G | 0.005 | . | . |
| **Intron 1** | **g.74220599A>G** | **rs8023330** | **G** | **0.75** | **0.42** | **<3.42x10^-4^** |
| Intron 1 | 74221005 | rs144884597 | G | 0.17 | . | . |
| Intron 1 | 74221074 | rs55899956 | C | 0.99 | 0.94 | 0.0012 |
| **Intron 1** | **g.74221157C>T** | **rs1550436** | **T** | **0.75** | **0.42** | **<3.42x10^-4^** |
| **Intron 1** | **74221298** | **rs1550437** | **C** | **0.97** | **0.82** | **<3.42x10^-4^** |
| **Intron 1** | **g.74221313G>C** | **rs6495085** | **G** | **0.99** | **0.82** | **<3.42x10^-4^** |
| Intron 1 | 74221492 | rs6495086 | C | 0.99 | 0.94 | 0.0012 |
| Intron 1 | 74221639 | rs1550439 | A | 0.02 | . | . |
| Intron 1 | 74221648 | rs117542509 | T | 0.01 | 0.01 | 0.87 |
| **Intron 1** | **g.74222028G>A** | **rs8034403** | **G** | **0.99** | **0.82** | **<3.42x10^-4^** |
| **Intron 1** | **g.74222043A>G** | **rs8034017** | **A** | **0.99** | **0.82** | **<3.42x10^-4^** |
| **Intron 1** | **g.74222202T>C** | **rs2165241** | **T** | **0.81** | **0.46** | **<3.42x10^-4^** |
| Intron 1 | 74222629 | ND | C | 0.005 | . | . |
| Intron 1 | 74222860 | ND | G | 0.005 | . | . |
| **Intron 1** | **g.74222987C>T** | **rs1078967** | **C** | **0.99** | **0.82** | **<3.42x10^-4^** |
| Intron 1 | 74223094 | ND | A | 0.005 | . | . |
| Intron 1 | g.74223118C>G | rs62004866 | G | 0.14 | 0.09 | 0.16 |
| **Intron 1** | **g.74223430C>G** | **rs28588430** | **C** | **0.82** | **0.45** | **<3.42x10^-4^** |
| Intron 1 | 74223478 | rs79999463 | G | 0.01 | 0.04 | 0.10 |
| Intron 1 | 74223507 | ND | A | 0.005 | . | . |
| **Intron 1** | **g.74223571T>C** | **rs28617339** | **T** | **0.82** | **0.45** | **<3.42x10^-4^** |
| **Intron 1** | **g.74223646G>T** | **rs74026308** | **G** | **0.99** | **0.82** | **<3.42x10^-4^** |
| **Intron 1** | **g.74223716G>C** | **rs28522673** | **G** | **0.99** | **0.82** | **<3.42x10^-4^** |
| **Intron 1** | **g.74223767C>G** | **rs1992314** | **C** | **0.84** | **0.64** | **<3.42x10^-4^** |
| Intron 1 | 74223885 | rs143717998 | A | 0.005 | 0.01 | 0.47 |
| Intron 1 | 74224631 | rs1440101 | A | 0.02 | . | . |
| **Intron 1** | **g.74224996G>A** | **rs4886776** | **G** | **0.84** | **0.64** | **<3.42x10^-4^** |
| **Intron 1** | **g.74225106G>A** | **rs8041642** | **G** | **0.99** | **0.82** | **<3.42x10^-4^** |
| **Intron 1** | **g.74225193G>A** | **rs8041685** | **G** | **0.99** | **0.82** | **<3.42x10^-4^** |
| Intron 1 | 74225270 | ND | G | 0.005 | . | . |
| **Intron 1** | **g.74225388C>A** | **rs4886778** | **C** | **0.83** | **0.45** | **<3.42x10^-4^** |
| **Intron 1** | **g.74225518C>T** | **rs8042039** | **C** | **0.99** | **0.82** | **<3.42x10^-4^** |
| Intron 1 | 74225667 | rs8027093 | C | 0.94 | 0.97 | 0.20 |
| Intron 1 | 74225704 | rs116089494 | C | 0.005 | 0.04 | 0.03 |
| Intron 1 | 74226023 | rs79768309 | A | 0.99 | 0.94 | 0.0012 |
| Intron 1 | 74226084 | rs77951661 | G | 0.99 | 0.94 | 0.0012 |
| **Intron 1** | **g.74226138G>A** | **rs8027022** | **G** | **0.83** | **0.45** | **<3.42x10^-4^** |
| **Intron 1** | **g.74226145G>A** | **rs58270968** | **G** | **0.84** | **0.64** | **<3.42x10^-4^** |
| Intron 1 | 74226433 | ND | A | 0.005 | . | . |
| Intron 1 | 74226470 | rs28603291 | T | 0.02 | . | . |
| **Intron 1** | **g.74226708C>G** | **rs2028386** | **C** | **0.83** | **0.45** | **<3.42x10^-4^** |
| **Intron 1** | **g.74226765G>C** | **rs4337252** | **G** | **0.83** | **0.45** | **<3.42x10^-4^** |
| Intron 1 | 74226901 | ND | T | 0.005 | . | . |
| Intron 1 | 74226902 | rs4243039 | G | 0.94 | 0.97 | 0.20 |
| Intron 1 | 74227013 | rs78246124 | C | 0.02 | 0.02 | 0.87 |
| **Intron 1** | **g.74227037A>C** | **rs2028387** | **A** | **0.84** | **0.64** | **<3.42x10^-4^** |
| Intron 1 | 74227140 | rs12594472 | T | 0.02 | . | . |
| Intron 1 | 74227478 | rs75616038 | T | 0.005 | 0.04 | 0.03 |
| Intron 1 | g.74227787C>T | rs72745362 | C | 0.96 | 0.91 | 0.06 |
| Intron 1 | g.74228391A>G | rs4077284 | G | 0.77 | 0.60 | 0.0004 |
| **Intron 1** | **g.74228464C>T** | **rs893816** | **C** | **0.84** | **0.64** | **<3.42x10^-4^** |
| Intron 1 | 74228602 | rs143653211 | A | 0.01 | . | . |
| **Intron 1** | **g.74228810G>A** | **rs4886782** | **A** | **0.52** | **0.33** | **<3.42x10^-4^** |
| Intron 1 | g.74229065G>A | rs893817 | A | 0.77 | 0.60 | 0.0006 |
| Intron 1 | 74229085 | ND | T | 0.005 | . | . |
| Intron 1 | 74229086 | ND | A | 0.005 | . | . |
| **Intron 1** | **g.74229195G>A** | **rs893818** | **G** | **0.84** | **0.64** | **<3.42x10^-4^** |
| Intron 1 | 74229403 | rs12917501 | G | 0.94 | 0.97 | 0.20 |
| **Intron 1** | **g.74229477G>A** | **rs67173550** | **G** | **0.84** | **0.64** | **<3.42x10^-4^** |
| Intron 1 | 74229490 | rs76675154 | A | 0.02 | . | . |
| Intron 1 | 74229510 | rs4423374 | G | 0.94 | 0.97 | 0.20 |
| **Intron 1** | **g.74229524A>G** | **rs893819** | **A** | **0.82** | **0.64** | **<3.42x10^-4^** |
| Intron 1 | 74229556 | ND | G | 0.02 | . | . |
| Intron 1 | 74229563 | rs4243040 | G | 0.94 | 0.97 | 0.20 |
| Intron 1 | 74229583 | ND | G | 0.005 | . | . |
| **Intron 1** | **g.74229603T>C** | **rs893820** | **T** | **0.82** | **0.64** | **<3.42x10^-4^** |
| Intron 1 | 74229678 | rs78839269 | A | 0.03 | . | . |
| Intron 1 | 74229781 | rs4243041 | C | 0.94 | 0.94 | 0.20 |
| **Intron 1** | **g.74229791G>A** | **rs4886785** | **G** | **0.84** | **0.64** | **<3.42x10^-4^** |
| Intron 1 | 74229957 | rs79631148 | A | 0.02 | . | . |
| Intron 1 | 74230117 | ND | A | 0.005 | . | . |
| Intron 1 | 74230199 | rs58490364 | A | 0.02 | . | . |
| Intron 1 | 74230356 | ND | A | 0.005 | . | . |
| Intron 1 | 74230550 | ND | A | 0.005 | . | . |
| **Intron 1** | **74230660** | **rs59755145** | **G** | **0.85** | **0.69** | **<3.42x10^-4^** |
| **Intron 1** | **g.74230806C>G** | **rs57783027** | **C** | **0.99** | **0.87** | **<3.42x10^-4^** |
| Intron 1 | 74231172 | ND | C | 0.005 | . | . |
| Intron 1 | g.74231179G>A | rs66481504 | G | 0.86 | 0.75 | 0.0084 |
| Intron 1 | 74231345 | rs202217795 | G | 0.01 | . | . |
| Intron 1 | 74231349 | rs16958494 | T | 0.02 | . | . |
| **Intron 1** | **g.74231439C>T** | **rs12440667** | **T** | **0.76** | **0.41** | **<3.42x10^-4^** |
| Intron 1 | 74231448 | ND | T | 0.85 | . | . |
| Intron 1 | g.74231451C>G | rs72745365 | C | 0.86 | 0.75 | 0.006 |
| Intron 1 | 74231503 | rs11855863 | C | 0.99 | 0.94 | 0.0012 |
| Intron 1 | g.74231733G>A | rs66497556 | G | 0.86 | 0.75 | 0.006 |
| **Intron 1** | **g.74231857T>C** | **rs9944214** | **C** | **0.92** | **0.67** | **<3.42x10^-4^** |
| Intron 1 | 74232049 | ND | C | 0.005 | . | . |
| **Intron 1** | **g.74232066A>G** | **rs72745367** | **A** | **0.97** | **0.70** | **<3.42x10^-4^** |
| Intron 1 | 74232365 | rs12594324 | T | 0.03 | . | . |
| **Intron 1** | **g.74232437G>A** | **rs12905253** | **A** | **0.76** | **0.41** | **<3.42x10^-4^** |
| Intron 1 | 74232497 | rs10851865 | A | 0.95 | 0.97 | 0.49 |
| Intron 1 | 74233480 | rs146322041 | G | 0.01 | . | . |
| Intron 1 | 74233751 | rs9783739 | A | 0.95 | 0.97 | 0.49 |
| Intron 1 | 74234059 | ND | A | 0.02 | . | . |
| **Intron 1** | **g.74234082C>G** | **rs11638944** | **G** | **0.76** | **0.41** | **<3.42x10^-4^** |
| Intron 1 | 74234136 | ND | T | 0.005 | . | . |
| Intron 1 | g.74234454A>T | rs139922201 | A | 0.99 | . | . |
| Intron 1 | 74234484 | rs11072449 | A | 0.95 | 0.96 | 0.69 |
| Intron 1 | 74234830 | ND | A | 0.005 | . | . |
| **Intron 1** | **g.74234902T>C** | **rs12441130** | **C** | **0.78** | **0.41** | **<3.42x10^-4^** |
| **Intron 2** | **g.74235500C>T** | **rs2304719** | **C** | **0.97** | **0.70** | **<3.42x10^-4^** |
| Intron 2 | 74235561 | ND | A | 0.005 | . | . |
| **Intron 2** | **g.74235704A>G** | **rs11631579** | **G** | **0.76** | **0.41** | **<3.42x10^-4^** |
| Intron 2 | 74235847 | rs28758267 | G | 0.86 | 0.75 | 0.006 |
| Intron 2 | 74235907 | rs12906558 | C | 0.79 | 0.71 | 0.08 |
| Intron 2 | g.74235979delT | rs59621340 | A | 0.88 | . | . |
| Intron 2 | 74236200 | ND | G | 0.005 | . | . |
| Intron 2 | 74236354 | ND | G | 0.005 | . | . |
| Intron 2 | 74236372 | rs7173840 | T | 0.95 | 0.97 | 0.32 |
| Intron 2 | 74236407 | rs7173508 | G | 0.95 | 0.97 | 0.32 |
| Intron 2 | 74236572 | ND | G | 0.005 | . | . |
| Intron 2 | 74236953 | rs1001507 | G | 0.99 | 0.94 | 0.0012 |
| Intron 2 | 74237028 | ND | G | 0.005 | . | . |
| Intron 2 | 74237120 | rs1530169 | C | 0.79 | 0.71 | 0.08 |
| Intron 2 | 74237381 | ND | T | 0.005 | . | . |
| Intron 2 | 74238020 | rs10775207 | A | 0.95 | 0.97 | 0.32 |
| Intron 2 | 74238272 | rs7163406 | G | 0.79 | 0.71 | 0.08 |
| Intron 2 | 74238683 | ND | A | 0.005 | . | . |
| **Intron 2** | **g.74238734T>C** | **rs2304720** | **T** | **0.97** | **0.72** | **<3.42x10^-4^** |
| Intron 3 | 74239253 | rs78826069 | G | 0.97 | 0.97 | 0.97 |
| Intron 3 | g.74239307G>A | rs67182653 | G | 0.87 | 0.77 | 0.01 |
| Intron 3 | 74239308 | rs142841160 | T | 0.02 | 0.02 | 0.82 |
| Intron 4 | 74239613 | rs74026313 | G | 0.99 | 0.94 | 0.0012 |
| Intron 4 | 74239869 | rs4595753 | T | 0.95 | 0.97 | 0.32 |
| Intron 4 | 74239871 | rs72745372 | C | 0.87 | 0.77 | 0.01 |
| Intron 5 | 74240354 | rs2304721 | C | 0.97 | 0.97 | 0.97 |
| Intron 5 | 74240537 | rs4497636 | G | 0.95 | 0.97 | 0.32 |
| Intron 5 | g.74241235T>C | rs893821 | T | 0.87 | 0.77 | 0.06 |
| **Intron 5** | **g.74241506G>A** | **rs750460** | **A** | **0.74** | **0.41** | **<3.42x10^-4^** |
| **Intron 5** | **g.74241624A>T** | **rs4243042** | **T** | **0.74** | **0.41** | **<3.42x10^-4^** |
| Intron 5 | 74241749 | rs2304722 | T | 0.87 | 0.77 | 0.06 |
| Intron 6 | g.74242067G>A | rs114910892 | A | 0.96 | 0.006 | 0.06 |
| Intron 6 | 74242383 | rs66473812 | T | 0.87 | 0.77 | 0.06 |
| **Intron 6** | **74243012** | **rs60579378** | **G** | **0.86** | **0.58** | **<3.42x10^-4^** |
| **Intron 6** | **g.74243246T>C** | **rs12437465** | **T** | **0.87** | **0.53** | **<3.42x10^-4^** |
| Intron 6 | 74243744 | rs11072450 | G | 0.81 | 0.70 | 0.07 |
| Intron 6 | 74243776 | ND | T | 0.01 | . | . |
| Intron 6 | 74243897 | rs12441138 | G | 0.96 | 0.98 | 0.32 |
| Exon 7 | 74244278 | rs8818 | C | 0.19 | 0.16 | 0,51 |
| Exon 7 | g.74244344C>T | rs3522 | C | 0.67 | 0.52 | 0.03 |
| 3´ Region | 74244592 | rs72745373 | C | 0.91 | 0.87 | 0.37 |
| 3´ Region | 74244610 | rs7173049 | A | 0.86 | 0.82 | 0.42 |
| 3´ Region | 74244824 | ND | A | 0.01 | . | . |
| 3´ Region | 74244855 | ND | T | 0.01 | . | . |
| 3´ Region | g.74245007T>G | rs7175324 | T | 0.65 | 0.45 | 0.0033 |
| 3´ Region | 74245296 | ND | T | 0.01 | . | . |
| 3´ Region | g.74245486C>T | rs12442139 | C | 0.65 | 0.47 | 0.0056 |
| 3´ Region | g.74245675G>A | rs12442211 | G | 0.65 | 0.47 | 0.0073 |
| 3´ Region | 74245837 | rs60563763 | T | 0.09 | 0.05 | 0.19 |
| 3´ Region | 74246045 | rs12913399 | C | 0.99 | 0.94 | 0.08 |
| 3´ Region | g.74246922A>G | rs12899085 | A | 0,50 | 0.35 | 0.02 |
| 3´ Region | 74247098 | rs117758648 | A | 0.03 | 0.02 | 0.92 |
| 3´ Region | 74247365 | rs117378025 | G | 0.92 | 0.92 | 0.88 |
| **3´ Region** | **g.74247468A>G** | **rs4886494** | **A** | **0.90** | **0.35** | **<3.42x10^-4^** |
| **3´ Region** | **g.74247471C>T** | **rs4886802** | **C** | **0.90** | **0.35** | **<3.42x10^-4^** |
| 3´ Region | 74247586 | rs148311323 | A | 0.03 | 0.02 | 0.76 |
| 3´ Region | 74247784 | rs11072451 | G | 0.83 | 0.77 | 0.22 |
| 3´ Region | g.74247896C>T | rs2415206 | C | 0.47 | 0.34 | 0.04 |
| 3´ Region | 74247931 | rs11072452 | G | 0.80 | 0.75 | 0.47 |
| 3´ Region | 74247935 | rs11631102 | A | 0.80 | 0.75 | 0.47 |
| 3´ Region | 74247944 | rs12904518 | A | 0.80 | 0.75 | 0.41 |
| 3´ Region | 74248360 | rs12102015 | T | 0.80 | 0.75 | 0.41 |
| 3´ Region | 74248388 | rs12101466 | A | 0.80 | 0.75 | 0.47 |
| 3´ Region | 74248464 | rs150438713 | T | 0.03 | 0.02 | 0.68 |
| 3´ Region | 74248477 | rs12102019 | T | 0.85 | 0.83 | 0.74 |
| 3´ Region | 74248548 | rs12101479 | G | 0.85 | 0.77 | 0.17 |
| 3´ Region | 74248604 | rs117625306 | A | 0.99 | 0.94 | 0.10 |
| 3´ Region | 74248864 | rs28642047 | A | 0.85 | 0.83 | 0.74 |
| 3´ Region | 74249642 | rs118128466 | A | 0.03 | 0.02 | 0.68 |
| 3´ Region | 74249856 | rs11072453 | A | 0.85 | 0.83 | 0.74 |
| 3´ Region | 74250338 | rs146742675 | G | 0.01 | 0.01 | 0.94 |
| 3´ Region | 74250427 | rs12907105 | G | 0.85 | 0.83 | 0.74 |
